# Nanocellulose/PEGDA Aerogels with Tunable Poisson’s Ratio Fabricated by Stereolithography for Mouse Bone Marrow Mesenchymal Stem Cell Culture

**DOI:** 10.3390/nano11030603

**Published:** 2021-02-28

**Authors:** Aimin Tang, Jiaoyan Ji, Jiao Li, Wangyu Liu, Jufang Wang, Qiuli Sun, Qingtao Li

**Affiliations:** 1 State Key Laboratory of Pulp and Paper Engineering, South China University of Technology, Guangzhou 510640, China; 201820126180@scut.edu.cn (J.J.); lijiao_fantong@163.com (J.L.); 2 School of Mechanical and Automotive Engineering, South China University of Technology, Guangzhou 510640, China; mewyliu@scut.edu.cn; 3 School of Bioscience and Bioengineering, South China University of Technology, Guangzhou 510640, China; jufwang@scut.edu.cn (J.W.); sunqiuli2013@163.com (Q.S.); mcqtli@scut.edu.cn (Q.L.)

**Keywords:** nanocellulose aerogels, stereolithography, Poisson’s ratio, chondrogenic induction

## Abstract

In this study, nanocellulose aerogels with a tunable Poisson’s ratio were fabricated. Tissue engineering scaffolds with a tunable Poisson’s ratio may be better able to simulate the mechanical behavior of natural tissues. A mixture of cellulose nanofibers (CNFs) and polyethylene glycol diacrylate (PEGDA) was used as the raw material to prepare CNF/PEGDA aerogels with a multiscale pore structure through a combination of stereolithography (SLA) and freeze-drying. The aerogels were fabricated with a regular macropore network structure and a random and homogeneous distribution of micropores. The macropore structure of the scaffolds could be customized through SLA, which resulted in scaffolds that exhibited one of three different mechanical behaviors: positive Poisson’s ratio (PPR), negative Poisson’s ratio (NPR) or zero Poisson’s ratio (ZPR). Then, the hydrogel scaffolds were transformed into aerogel scaffolds through the freeze-drying method, which endowed the scaffolds with homogeneously distributed micropores. The material ratio and exposure were adjusted to obtain scaffolds with a clear pore structure. Then, the CNF/PEGDA scaffolds with different Poisson’s ratios were subjected to mechanical tests, and their chondrogenic induction characteristics were determined. The NPR scaffold not only provided a good environment for cell growth but also affected mouse bone marrow mesenchymal stem cell (mBMSC) proliferation and chondrogenic induction. Thus, we provide a feasible scheme for the preparation of three-dimensional scaffolds with a multiscale pore structure and tunable Poisson’s ratio, which contributes to cartilage repair in tissue engineering.

## 1. Introduction

Scaffolds are an important part of tissue engineering research and can provide a good environment for cell growth, cell proliferation, cell differentiation and tissue formation [1,2]. Based on in-depth research in the field of tissue engineering, ideal tissue engineering scaffolds need biocompatibility, a porous structure and appropriate mechanical properties [1,2,3,4]. Moreover, scaffolds should simulate the mechanical behavior that natural tissues exhibit under external forces [5]. Poisson’s ratio can be used to describe the ability of scaffolds to support and transmit cell and tissue forces in tissue engineering. It refers to the ratio of the absolute value of transverse normal strain and axial normal strain when a material is under uniaxial tension or compression [6]. Due to unique deformation mechanisms, materials with different Poisson’s ratios have different mechanical effects. Generally, materials with different Poisson’s ratios have different pore structures, including the shape and size of pores. However, different biological tissues often have different structural shapes and sizes and exhibit different Poisson’s ratio effects under the action of external forces. For example, the Poisson’s ratio of heart valvular and cardiovascular tissue is negative [7]. The Poisson’s ratio of cartilage is close to zero [8]. Scaffolds with a tunable Poisson’s ratio have been reported to be suitable for cartilage repair because they can not only imitate the growth environment of chondrocytes but also affect the proliferation of cells under the stimulation of the external environment [9,10,11]. Besides, Song et al. found that scaffolds with a negative Poisson’s ratio could promote the expression of vascular differentiation markers and the secretion of the extracellular matrix (ECM) protein vitronectin [12]. Therefore, the Poisson’s ratio of scaffolds can be adjusted to match the properties of the target tissue.

The Poisson’s ratio of scaffolds can be adjusted through the pattern design of the pore structure. If the deformation of the scaffold material remains elastic, the Poisson’s ratio is controlled exclusively by the pore structure [8,10]. The structures of conventional hexagonal honeycombs, re-entrant honeycombs and semi re-entrant honeycombs can exhibit positive Poisson’s ratios (PPRs), negative Poisson’s ratios (NPRs) and zero Poisson’s ratios (ZPRs), respectively [13]. Kapnisi et al. fabricated a chitosan-polyaniline cardiac patch with a re-entrant honeycomb structure by excimer laser microablation [14]. Ex vivo studies demonstrated that cardiac patches with a negative Poisson’s ratio conformed better to cardiac movements than unpatterned patches of the same material. Lee et al. fabricated polyethylene glycol diacrylate (PEGDA) scaffolds that had either NPR or PPR by projection stereolithography and demonstrated that cell attachment was increased in scaffolds that had NPR [7]. Soman et al. prepared PEGDA scaffolds with tunable Poisson’s ratio through Mask prototyping stereolithography [10]. Strain measurement results showed that the hybrid PEGDA scaffolds exhibited both NPR and PPR behavior, and the scaffolds were applied to a culture of human mesenchymal stem cells. In current studies, although the Poisson’s ratio of scaffolds is regulated by grid design, it is difficult to obtain scaffolds with multistage pore structures. However, in tissue engineering, a multistage pore structure plays an important role in cell adhesion, nutrient transport and waste discharge. Lien et al. showed that micropores with a diameter of 250–500 μm promote chondrocyte proliferation and extracellular matrix (ECM) generation, whereas nanoscale pores with a diameter of 50–200 μm promote cell differentiation [1,15]. Macropores can be obtained using a grid design, whereas micropores or nanoscale pores are obtained by combining other manufacturing methods. Therefore, it is necessary to prepare aerogels with different Poisson’s ratios to meet the requirements for simulating the mechanical behavior of natural tissues and cell culture.

Aerogels are ultra-light materials with high porosity and specific surface area. The liquid components in a wet-gel precursor can be sublimated by critical-point drying (CPD) or lyophilization (freeze-drying) to fabricate porous materials [16,17,18]. In tissue engineering, porous aerogels are prepared to permit the proper exchange of waste or nutrients for growing cells. Due to their biodegradability, biocompatibility and high surface area, cellulose nanofibers (CNFs) have been widely studied and applied to the synthesis of biocompatible aerogels [19]. CNF aerogels have been demonstrated to enhance cell growth and proliferation [20,21]. Nanocellulose-based scaffolds have been researched for ligament, skin and cartilage tissue engineering [22,23,24]. For the purpose of cartilage tissue engineering, Naseri et al. fabricated freeze-dried CNF scaffolds that could retain viable chondrocytes for up to 7 days [4]. Current research has enabled scaffolds to simulate the natural chemistry and morphology of different areas of articular cartilage [24]. However, nanocellulose aerogels with different Poisson’s ratios and the effects of the Poisson’s ratios on cell growth and differentiation have not been studied.

Stereolithography (SLA) can be applied to accurately customize the structure of three-dimensional tissue engineering scaffolds [25,26]. Liquid photocurable resins serve as the ink in 3D printers and can be photopolymerized to form hydrogels with specific structures based on digital design patterns upon exposure to UV light. According to the principle of SLA, the raw materials used in the photocuring process are mostly photocurable resins. However, CNFs lack photocuring properties to form shapes, and thus, fabrication of CNF nanocomposite hydrogels for tissue engineering via SLA remains a challenge. Napolabel et al. demonstrated that the incorporation of cellulose nanocrystals into a PEGDA matrix could allow fabrication of 3D-printed nanocomposite hydrogels through SLA to improve different properties of hydrogels [25]. Considering the role of Poisson’s ratio in tissue engineering, Soman et al. made hybrid PEGDA scaffolds with a specific Poisson’s ratio to culture stem cells [8,10], but the scaffolds were unable to meet the requirements of cell growth and differentiation due to the lack of a multiscale porous structure. Thus, to provide a good space for cell growth, we proposed a project combining the advantages of SLA and freeze-drying to prepare porous nanocellulose aerogel scaffolds with different Poisson’s ratios.

In our previous study, columnar hydrogels were initially fabricated via SLA with the incorporation of PEGDA into a CNF matrix as raw materials [27]. Li et al. fabricated columnar nanocellulose aerogel scaffolds with a uniform pore distribution and porosity of 90% by combining SLA and freeze-drying [28]. Subsequently, nanocellulose hydrogels with tunable Poisson’s ratios were successfully prepared by designing three different honeycomb structures. The honeycomb structure was designed by AutoCAD. Then, after optical printing, masks with different structural patterns were obtained. Finally, with Irgacure 2959 as the photoinitiator, the liquid-curable resin was photopolymerized under ultraviolet light irradiation to fabricate hydrogels with a honeycomb structure [29]. Although hydrogels with a tunable Poisson’s ratio have been successfully prepared, they have not yet been applied as biological scaffolds in tissue engineering because the hydrogel aperture size is too large to be suitable for cell culture.

In the present study, to obtain scaffolds with a tunable Poisson’s ratio, we designed a specific grid structure for scaffolds by AutoCAD. Then, CNFs and PEGDA were used as the printing ink for SLA. In the photocuring process, the material ratio and the exposure time were adjusted to obtain CNF/PEGDA hydrogels. After freeze-drying, CNF/PEGDA aerogels with a multiscale pore structure and a tunable Poisson’s ratio were fabricated. As a photocurable resin, the PEGDA could be cross-linked, which endowed the hydrogels with customized pore structures, and the nanocellulose contributed significantly to micropore formation in the aerogels after freeze-drying. The macrostructures and microstructures of the CNF/PEGDA scaffolds were observed, and the compression modulus was analyzed. Moreover, the effects of the Poisson’s ratio on the proliferation and chondroblast differentiation of mouse bone marrow mesenchymal stem cells (mBMSCs) were studied. This work contributes to the culture and functional induction of stem cells in tissue engineering.

## 2. Materials and Methods

### 2.1. Materials and Reagents

A CNF suspension (2 wt%) was prepared in our laboratory. The 2,2,6,6-Tetramethylpiperidine-1-oxyl (TEMPO), the photocurable resin PEGDA, Irgacure 2959 and dimethyl sulfoxide were purchased from Sigma-Aldrich (Shanghai, China) Trading Co., Ltd. NaClO at a concentration of 10% was purchased from Tianjin Fuyu Fine Chemicals Co., Ltd. (Tianjin, China). NaBr was purchased from Tianjin Kermel Chemical Reagent Co., Ltd. (Tianjin, China). C57BL/6 mBMSCs were provided by the School of Materials Science and Engineering at South China University of Technology (Guangzhou, China). C57BL/6 mouse bone marrow mesenchymal stem cell complete culture medium and C57BL/6 mouse bone marrow mesenchymal stem cells into chondrocytes induced differentiation of complete medium and toluidine blue stain were purchased from Saiye (Suzhou, China) Biological Information Technology Co., Ltd. An MTT assay kit, a calcein-AM (C-AM)/propidium iodide (PI) double-staining kit and tolonium chloride, used in the detection process, were obtained from Shanghai Yisheng Bio-Technology Co., Ltd. A hematoxylin and eosin (HE) staining kit was purchased from Beijing Leigen Biotechnology Co., Ltd. Trypsin solution was purchased from Beijing Bioasentai Biotechnology Co., Ltd. Phosphate-buffered saline (PBS) (Helicus) was purchased from Shanghai Shuangluoxuan Biological Technology Co., Ltd.

### 2.2. Preparation of CNF Suspensions

CNFs were prepared by TEMPO/NaBr/NaClO alkaline medium oxidation of bleached eucalyptus pulp combined with ultrasound wave treatment [30]. First, the bleached eucalyptus pulp was oxidized by the TEMPO/NaBr/NaClO alkaline oxidation system (the TEMPO, NaBr and NaClO concentrations were 1.0, 10 and 8 mmol/g, respectively), and then, oxidized pulp with a carboxyl content of 1.37 mmol/g was obtained. After that, the oxidized pulp suspension was treated with an ultrasonic wave disrupter (Guangzhou Newpower Ultrasonic Electronic Equipment Co., Ltd.) at an ultrasonic frequency of 15 kHz for a working time of 90 min; thus, a CNF suspension with a carboxyl content of 1.37 mmol/g was obtained.

### 2.3. Structural Design of Tissue Engineering Scaffolds

**Figure 1:**
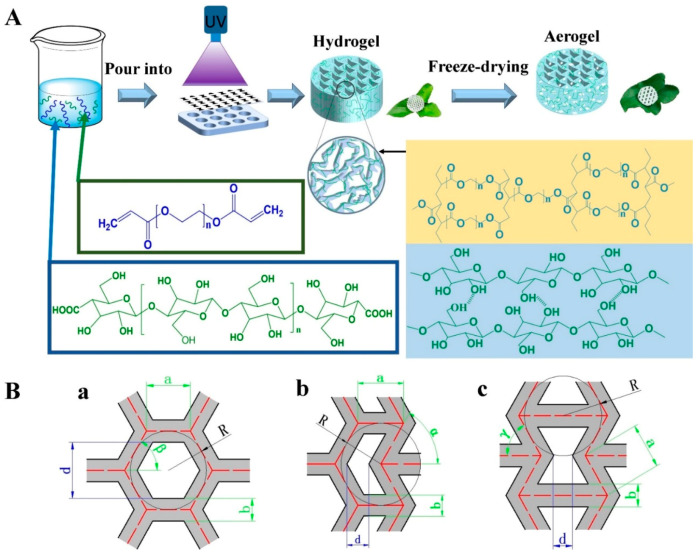
(**A**) Schematic flow diagram showing CNF/PEGDA aerogel scaffold preparation. (**B**) Geometric blueprints for three different Poisson’s ratio structures: (**a**) positive Poisson’s ratio (PPR); (**b**) negative Poisson’s ratio (NPR); (**c**) zero Poisson’s ratio (ZPR).

Three types of scaffolds with different pore structures were designed using AutoCAD (Figure 1B): scaffolds with a PPR, ZPR and NPR. The honeycombs had a PPR structure, and the re-entrant honeycombs had an NPR structure. A ZPR was achieved by another variant of the honeycomb model known as a semi-re-entrant honeycomb structure [15,31]. The unit geometry of the different Poisson’s ratio structures and the relevant dimensional parameters are presented in Table 1 (angles are all 60°). Then, the designed unit geometry was optically printed to construct a film mask for the experiment (Figure 1B).

### 2.4. Preparation of CNF/PEGDA Photocurable Resin Mixtures

CNF suspensions were freeze-dried to obtain CNF aerogels. A certain amount of CNF foam was first shredded into small pieces and then slowly added to deionized water. The CNF suspensions were bathed at 35 °C and stirred with magnetic force for 20 min. An ultrasonic cell crusher was used for ultrasonic dispersion treatment for 30 s with a power of 100 W. High concentrations of CNF redispersible suspensions (2 wt%) were obtained by stirring in a water bath at 35 °C for 10 min. Certain amounts of PEGDA and photoinitiator were added and mixed with the CNF suspension. Then, the CNF/PEGDA resin was placed in a water bath and magnetically stirred at 35 °C for 20 min. The CNF/PEGDA resin formulas are listed in Table 2. Then, the CNF/PEGDA mixture was treated by an ultrasonic cleaner to remove the bubbles in the mixture. Finally, a uniform and stable CNF/PEGDA resin was obtained and stored in a cool and dark environment.

### 2.5. Fabrication of CNF/PEGDA Scaffolds

The CNF/PEGDA photocurable resin mixtures were injected into a polytetrafluoroethylene board. Under a UV intensity of 11 MW/cm^2^, CNF/PEGDA hydrogels with different Poisson’s ratios were fabricated by covering the film mask over UV-curing mixtures. Then, the hydrogel was pre-frozen in a refrigerator at −20 °C for 24 h and freeze-dried in a freeze-dryer at −68 °C for 24 h. Finally, CNF/PEGDA aerogel scaffolds were fabricated. A schematic of the fabrication of the CNF/PEGDA aerogel scaffolds is shown in Figure 1.

### 2.6. Analysis of the Clarity of CNF/PEGDA Hydrogel Structure Formation

An optical microscope (OLYMPUS BX51; Olympus Corporation, Shinjuku, Tokyo) and digital camera (EOS M50; Canon Corporation, Tokyo, Japan) were used to observe the appearance and structural outlines of the CNF/PEGDA hydrogels.

### 2.7. Macromorphology and Micromorphology of CNF/PEGDA Scaffolds

A digital camera was used to observe the macroscopic morphology of CNF/PEGDA scaffolds. To observe the microstructure of the scaffolds, the hydrogels were placed in a frozen slicer and pre-frozen for 5 min to form a jelly. To obtain samples, the jelly was sliced with a blade in a freezing microtome. The samples were freeze-dried and then observed using an EVO18 field emission scanning electron microscope (Carl Zeiss Jena, Oberkochen, Germany) under ultra-high vacuum conditions with an accelerating voltage of 10.0 kV.

### 2.8. Compressive Test of CNF/PEGDA Scaffolds

The compression modulus of CNF/PEGDA scaffolds with a 15 mm diameter and 3-mm height was tested using an Instron-5565 universal material testing machine. The sensor was set at 100 N and the compression rate was 0.5 mm/min.

### 2.9. Stem Cell Culture and Differentiation on CNF/PEGDA Scaffolds

Sixth-generation mBMSCs were extracted from cell culture dishes by using trypsin and prepared into cell suspensions. The aerogels were sterilized with ethylene oxide and placed in a 24-well plate. Then, the stem cells were seeded on the aerogel material at a density of 4 × 10^4^ per well. After the cell suspension was fully absorbed by the material, complete culture medium was added at 500 μL. Then, the cells/scaffolds were cultured in a cell incubator, and the stem cell culture medium was changed every other day. After three days, part of the pore plates was discarded from the old stem cell medium and added to the chondroblast differentiation culture medium as the induction group. Others were treated with the original full medium as a non-induced group. Finally, both groups of cell/scaffold cultures were placed in a tube at 37 °C and 5% CO_2_. The medium was changed every night for 21 days.

### 2.10. SEM Analysis of Cell–Scaffold Constructs

After 3 days of stem cell culture, the old culture medium was discarded, and the cell scaffolds were washed twice with PBS and fixed with 2.5% (V/V) glutaraldehyde for at least 5 h. On the second day, the fixed cell–scaffold constructs were rinsed with PBS 6 times for 20 min each time. Then, the cell scaffolds were rinsed with 30% (V/V) alcohol twice for 10 min each time and rinsed with 50% (V/V) alcohol twice for 10 min each time. Finally, they were rinsed with 70% (V/V) alcohol for 15 min and stored overnight. On the third day, 90% (V/V) alcohol was used to flush the scaffolds for 10~15 min. Then, the scaffolds were rinsed thrice with anhydrous acetone plus anhydrous calcium chloride for 10~15 min each time. Finally, each freeze-dried cell/aerogel complex was observed via field emission scanning electron microscopy (Hitachi S-3000N, Hitachi Ltd., Tokyo, Japan) to evaluate the morphology of cells in the aerogels.

### 2.11. CLSM Analysis of Cell–Scaffold Constructs

To observe the stem cells cultured on scaffolds for 3 days and the cellular activity of chondrocytes induced for 3 weeks, the cell scaffolds were stained with C-AM and PI, and then, images of the cell scaffolds were obtained via confocal laser scanning microscopy (CLSM; Carl Zeiss LSM-700, Leica TCS-SP5, Leica Microsystems, Wetzlar, Germany). First, the cell scaffolds were washed twice with PBS. Second, 500 µL of C-AM/PI dye (2 M C-AM, 4.5 M PI) was added to each well, and the cell scaffolds were incubated in a constant-temperature incubator for 20 min. Finally, the stained cell scaffolds were washed twice with PBS, and the cell viability was observed by CLSM at excitation wavelengths of 490 and 515 nm to observe C-AM and PI staining, respectively; the emission wavelengths were 535 and 617 nm, respectively.

### 2.12. MTT Analysis of Cell–Scaffold Constructs

The viability of stem cells cultured for 1, 3, 5 and 7 days was measured using the MTT method. The cells/scaffolds that were obtained from a 24-well plate were placed into a new 24-well plate and washed twice with PBS. Then, 400 μL MTT solution (5 mg/mL) was added to each well of the 24-well plate. The culture plate was incubated for 4 h at 37 °C in an atmosphere of 5% CO_2_. Four hours later, the cell scaffolds were placed into 96-well plates and shaken with dimethyl sulfoxide for 10 min. The optical density (OD) values were measured by a microplate reader (SpectraMax M5) at a wavelength of 570 nm. Finally, the OD value of each well in the 96-well plate was converted into the OD value of each well in the 24-well plate.

### 2.13. Staining Analysis of Cartilage Matrix Secretion

Cell scaffolds that were induced for 14 days were stained with alcian blue and toluidine blue to detect proteoglycan secretion by chondroblasts and glycosaminoglycan secretion in the chondroblast matrix. First, the cell scaffolds were washed with PBS for 5 min, fixed with 4% paraformaldehyde for 30 min and then washed with PBS twice for 5 min each time. The cell scaffolds were cut into thin flakes with a freezing microtome and adhered to glass slides. Next, the samples were stained with alcian blue for 30 min and washed with distilled water for 2 min to stop staining, and the excess dye was washed away from the samples. Then, the samples were dehydrated in a concentration gradient of 70%, 80%, 90%, 95% and 100% ethanol for 1 min at each concentration; cleaned with a tissue transparency agent twice for 1 min each time; dripped with neutral gum; covered with a cover glass and dried at 30 °C. Finally, the staining results were observed via optical microscopy (OLYMPUS BX51; Olympus Corporation, Shinjuku, Tokyo).

### 2.14. HE Staining of Cell–Scaffold Constructs

To detect cell morphology, cell scaffolds that were induced for 21 days were stained with HE. The cell scaffolds were rinsed with PBS for 5 min, fixed with 4% paraformaldehyde for 30 min, washed with PBS twice for 5 min each time, cut into thin flakes with a freezing microtome and adhered to glass slides. The samples were first dyed with hematoxylin for 30 min and washed with distilled water for 2 min to stop staining, and then, the excess dye was washed away from the samples. Second, after differentiation by addition of acetic acid for 30 s, the samples were washed with distilled water for 5 min, stained with eosin for 2 min and cleaned with distilled water for 2 min. The samples were then dehydrated in a concentration gradient of 70%, 80%, 90%, 95% and 100% ethanol for 1 min at each concentration; cleaned with a tissue transparency agent twice for 1 min each time; dripped with neutral gum; covered with a cover glass and dried at 30 °C. Finally, the staining results were observed via optical microscopy (OLYMPUS BX51; Olympus Corporation, Shinjuku, Tokyo).

## 3. Results and Discussion

### 3.1. Fabrication of CNF/PEGDA Hydrogels

According to the structures and sizes shown in Figure 1B and Table 1, the scaffold structures were drawn using AutoCAD, and the corresponding films were generated via optical printing. The CNFs and initiator were evenly dispersed in PEGDA solution to produce a homogeneous ink, and then, CNF/PEGDA hydrogels with customized pore structures were fabricated by SLA. Generally, under UV irradiation, the acrylate terminal groups of the linear macromolecule PEGDA covalently cross-link to form a PEGDA macromolecule network in which nanocellulose is wrapped, leading to the formation of a CNF/PEGDA hydrogel.

**Figure 2:**
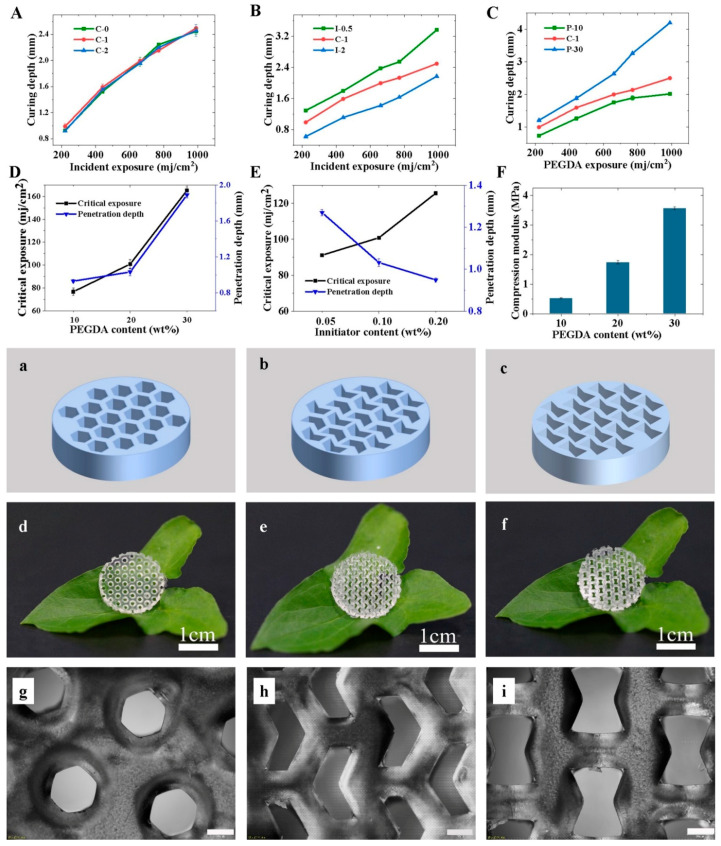
Adjustment of the material ratio of the CNF/PEGDA-blend photocurable resin during the photocuring process. (**A**–**C**) Relationship between curing depth and incident exposure for blended photocurable resins with different material ratios: (**A**) CNF content; (**B**) initiator content; (**C**) PEGDA content. (**D**–**E**) Effect of initiator content and PEGDA on the critical exposure (Ec) and penetration depth (Dp) of CNF/PEGDA blended photocurable resins: (**D**) CNF content 1% and PEGDA content 20 wt%; (**E**) CNF content 1% and initiator content 0.1 wt%. (**F**) Compression modulus of CNF/PEGDA hydrogel at different PEGDA content levels. (**a**–**f**) Ideal and actual CNF/PEGDA hydrogels: (**a**–**c**) ideal hydrogels; (**d**–**f**) actual hydrogels. (**g**–**i**) Optical microscopy of CNF/PEGDA hydrogel; the scale is 500 μm. Left to right: (**a**,**d**,**g**) PPR; (**b**,**e**,**h**) ZPR; (**c**,**f**,**i**) NPR.

To obtain ideal CNF/PEGDA hydrogels (Figure 2) with a mechanical strength that satisfies the necessary requirements, the effects of the CNF/PEGDA-blend photocurable resin components (including CNFs, initiator and PEGDA) on the curing depth (Cd) of the blended photocurable resin were studied to optimize the material ratio of the blended photocurable resin. The relationship between curing depth and incident exposure for CNF/PEGDA-blend photocurable resins with different material ratios is shown in Figure 2A–C.

As shown in Figure 2, the Cd of the blend resin was independent of the CNF content and was only affected by the incident exposure. The initiator and PEGDA act as curing components in the blended photocurable resin system. Critical exposure (Ec) and penetration depth (Dp) are the basic characteristic parameters of photocuring resins. Considering the influence of the initiator and PEGDA on the formation and compressive modulus of hydrogels, the initiator dose and PEGDA dose for the blended photocurable resin were 0.1 wt% and 20 wt%, respectively.

To fabricate CNF/PEGDA hydrogels with the desired pore structure in accordance with the experimental design requirements, the exposure was adjusted to ensure that the CNF/PEGDA hydrogels had clear structural outlines. The exposure is the product of curing time and incident exposure. As shown in Figure S1, the optimal curing durations for PPR, NPR and ZPR were approximately 60, 65–70 and 70–75 s, respectively. Finally, we obtained the thicknesses of the PPR hydrogel, the ZPR hydrogel and the NPR hydrogel, as shown in the figure, of 1.46, 1.49 and 1.51 mm, respectively.

MATLAB was used to simulate the actual curing experiment. The degree of solidification was different under the opaque portion, and the solidified patterns were circular and had alternate rules. In addition, the distribution of light intensity on the resin surface changed uniformly due to diffraction. This finding explains the excessive curing of the inner corner edges of different honeycomb structures in SLA. Therefore, this photocuring method is not suitable for preparing thick hydrogels.

### 3.2. Fabrication of Variable Poisson’s Ratio CNF/PEGDA Aerogel Structures

**Figure 3:**
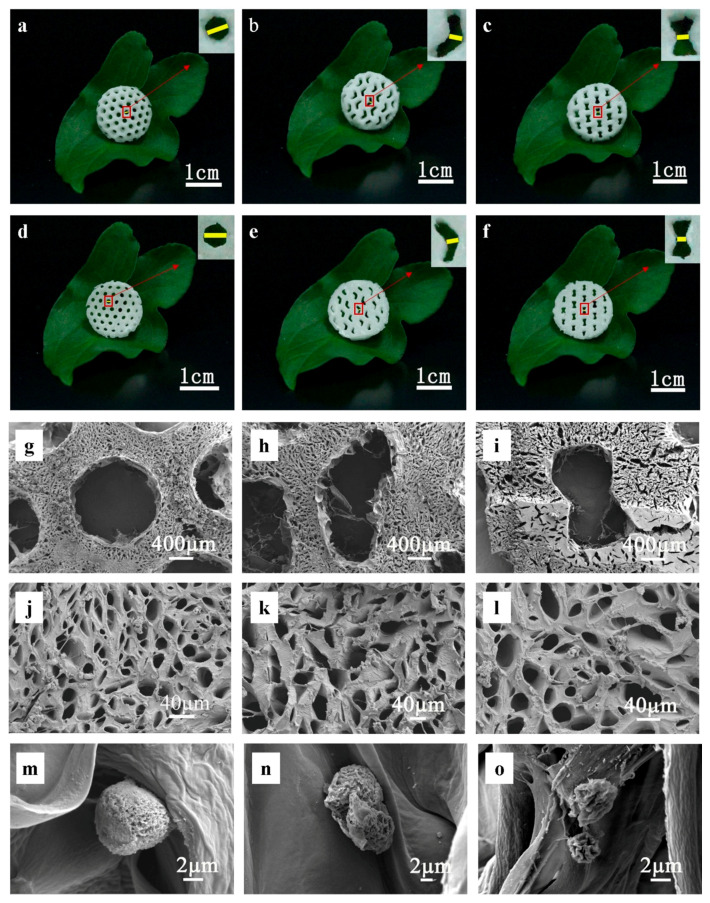
(**a**–**f**) Digital photographs of the exposure surface and backlit surface of CNF/PEGDA aerogels. (**g**–**l**) SEM images of CNF/PEGDA scaffolds with different Poisson’s ratios. (**m**–**o**) SEM images of cell adhesion after inoculation on CNF/PEGDA scaffolds. Left to right: (**a**,**d**,**g**,**l**,**m**) PPR; (**b**,**e**,**h**,**k**,**n**) ZPR; (**c**,**f**,**i**,**l**,**o**) NPR. The illustration shows a magnified view of local apertures.

According to the material ratio and exposure values determined above, CNF/PEGDA hydrogels with different honeycomb structures were prepared by SLA. The hydrogels were then freeze-dried to obtain aerogel scaffolds (Figure 3A). CNFs in hydrogels play an important role in the freeze-drying process [18]. CNF/PEGDA hydrogels exhibit uniform CNF dispersion, and after the sublimation of water molecules, they form stable nanocellulose structures called nanocellulose aerogels [32].

The morphology of the CNF/PEGDA aerogels was assessed with a digital camera to observe the aerogels prepared with the honeycomb mask. In Figure 3a–f, the CNF/PEGDA aerogels with different Poisson’s ratios all have inerratic pore structures. The results show that the combination of SLA and freeze-drying can be applied to obtain nanocellulose-based scaffolds with multiscale pore structures. This finding demonstrates the feasibility of the experimental scheme.

The microporous structure and pore wall of CNF/PEGDA scaffolds are shown in Figure 3g–l. The nanocellulose aerogels not only have regular macropores with sizes between 400 and 800 μm but also a homogeneous distribution of micropores, and the micropore size ranges from 20 to 100 μm. This aperture range can ensure the passage of ordinary cells and provide sufficient space for cell growth and migration [33]. In addition, this aperture size is sufficiently small to prevent cells from draining directly from the scaffold during culture.

In Figure 3, protrusions can be noted on the macropore wall of the CNF/PEGDA aerogel. These filaments and protrusions increase the specific surface area of the pore wall to a certain extent, providing more attachment points for cells in the tissue engineering scaffold and improving the cell inoculation efficiency of the scaffold. The formation of these filaments may result from the self-assembly behavior of CNFs at the edge of the macropores during the formation of ice crystals during freeze-drying [17]. mBMSCs adhered to the pore wall of the scaffold in the form of single cells, as shown in Figure 3m–o, which were round in shape, with a cell size of 2~8 μm. PEG hydrogels have been reported to be too smooth for cell adhesion [34].

The addition of nanocellulose increased the specific surface area of the composite scaffolds. The microporous wall of the scaffolds was uneven with a nano-level roughness. Pore walls with nanoscale roughness provide attachment points for cells and may be favorable for cell interactions [4]. To further study the role of the pore structure of scaffolds with different Poisson’s ratios, we conducted several experiments to analyze stem cell proliferation and differentiation on aerogel scaffolds.

### 3.3. Compression Modulus of CNF/PEGDA Scaffolds

**Figure 4:**
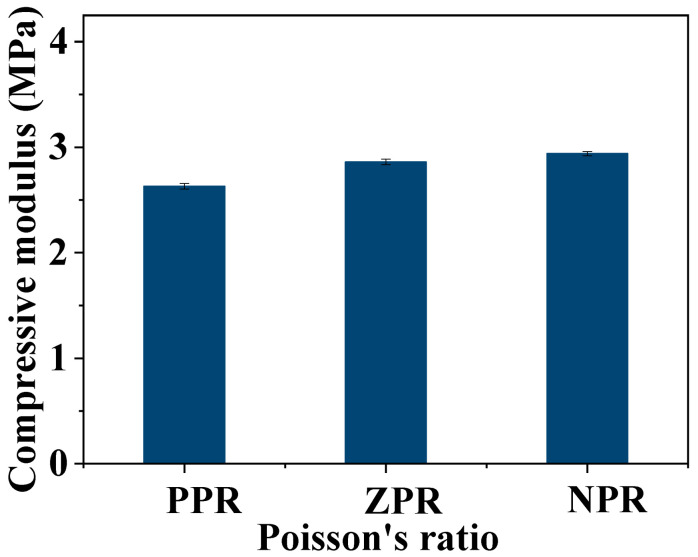
Compression modulus of CNF/PEGDA aerogels with different Poisson’s ratios.

To study the mechanical properties of different honeycomb structures, compression tests of aerogel scaffolds with different Poisson’s ratios were conducted, and the results are shown in Figure 4. The compression moduli of the PPR, ZPR and NPR CNF/PEGDA aerogels were 2.63, 2.86 and 2.94 MPa, respectively.

### 3.4. Survival Rate Analysis of Stem Cells on CNF/PEGDA Scaffolds

**Figure 5:**
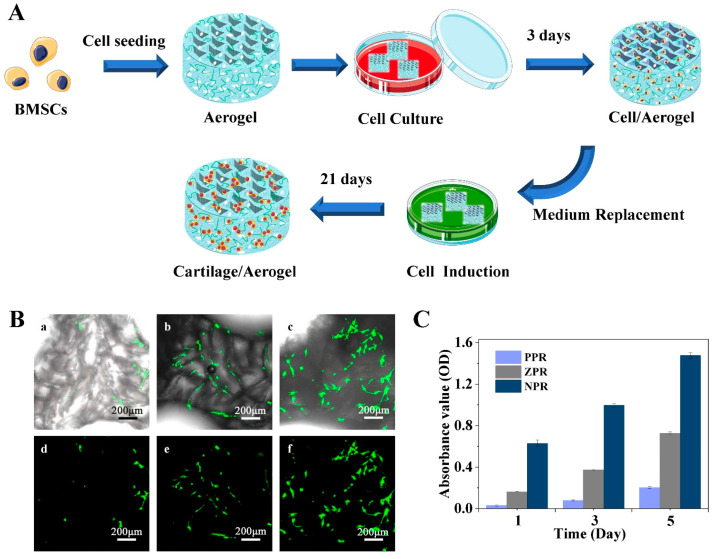
(**A**) Schematic diagram of mouse bone marrow mesenchymal stem cells (mBMSCs) cultured and induced on CNF/PEGDA scaffolds. (**B**) Confocal laser scanning microscopy (CLSM) images of mBMSCs cultured for 3 days in CNF/PEGDA aerogels with different Poisson’s ratios: (**a**,**d**) PPR; (**b**,**e**) ZPR; (**c**,**f**) NPR. (**C**) Proliferation of mBMSCs cultured for 1, 3 and 5 days in CNF/PEGDA aerogels with different Poisson’s ratios.

To evaluate the viability of cells cultured on the scaffolds, CLSM and MTT tests were used to observe the live/dead cell numbers and cell proliferation on scaffolds cultured for 3 days. Cell-reactive dyes (C-AM and PI) were used to stain the cell scaffolds; live cells were stained green by C-AM, and dead cells were stained red by PI. In Figure 5B, green live mBMSCs accounted for the majority, and almost no red dead cells were found. This result indicates that mBMSCs cultured on CNF/PEGDA aerogel scaffolds for 3 days may still exhibit good cellular activity. Figure 5B shows that the cells spread rather than aggregating along the edge of the macropore, which indicates that the CNF/PEGDA aerogels have interconnected pores and high scaffold permeability to convey sufficient nutrients for stem cells [35]. The number of cells on the NPR aerogel scaffolds was the highest, followed by the ZPR scaffolds, whereas the number on the PPR scaffolds was the lowest.

To further study the growth of mBMSCs on scaffolds with different Poisson’s ratios, MTT tests were used to determine the proliferation of mBMSCs on days 1, 3 and 5 after cell seeding on aerogel scaffolds, and the results are shown in Figure 5C. MTT reagents can interact with live cells to form blue-colored formazan crystals. The OD value of the formazan crystals was determined using a microplate reader at a wavelength of 570 nm [28]. As shown in Figure 5C, the OD value increased with culture time, indicating that the mBMSCs grew and multiplied with culture time. Compared with the other two types of honeycomb scaffolds, the NPR aerogel scaffolds had the maximum light absorption value at different culture times, indicating that NPR scaffolds could provide a suitable growth environment for stem cells.

### 3.5. Analysis of Stem Cell Induction on CNF/PEGDA Scaffolds

To study the induced differentiation effect of mBMSCs on CNF/PEGDA scaffolds with different Poisson’s ratios, non-induced and induced cells/scaffolds were stained to observe the induction effect.

**Figure 6:**
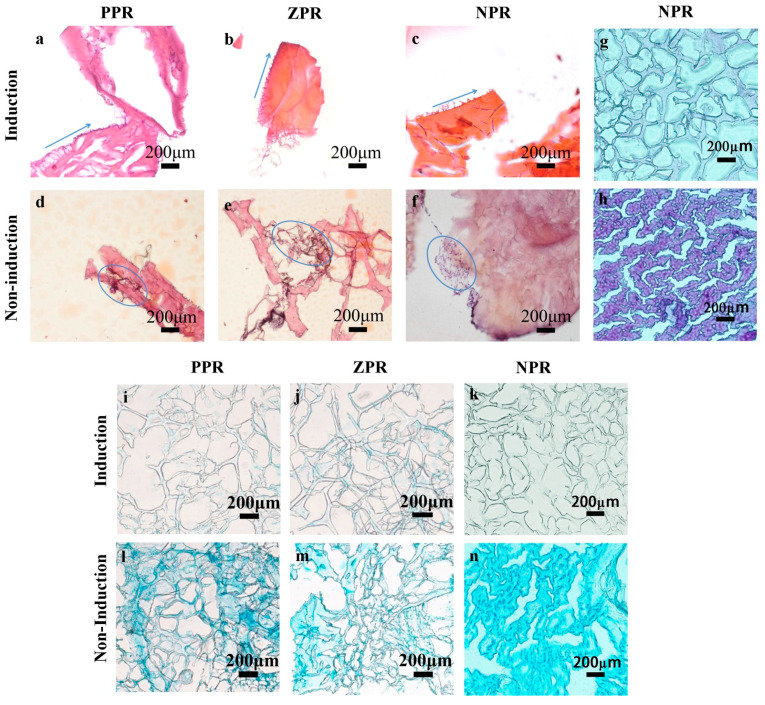
(**a**–f) Hematoxylin and eosin (HE) staining of mBMSCs induced on different Poisson’s ratio CNFs/PEGDA scaffolds for 3 weeks. (**g**–**n**) Staining of cartilage matrix of mBMSCs after 2 weeks of non-induction and induction on the NPR CNFs/PEGDA scaffolds. (**g**–**h**) Toluidine blue staining of mBMSCs; (**i**–**n**) Alcian blue staining of mBMSCs.

To investigate whether markers of cartilage matrix were generated, the cartilage matrix in cell scaffolds was stained, as shown in Figure 6. Alcian blue and toluidine blue can be used as specific dyes to identify chondrocytes, because alcian blue stains characteristic markers of hyaline cartilage called proteoglycans (PGNs) and toluidine blue stains chondrocyte stromal glycosaminoglycan (GAG). The staining results revealed that the color intensity in the induced group was darker than that in the non-induced group. Although the non-induced group showed slight staining due to adsorption of the dye to the aerogel material, the staining of chondrocyte aggregation sites was significantly enhanced. As shown in Figure 6g–h, it was found that the cartilage matrix of the NPR scaffold was stained with toluidine blue, which was darker than that of the non-induced group. Furthermore, after Alcian blue staining of the scaffolds with different Poisson’s ratio, the NPR scaffolds were stained more significantly than the other two scaffolds in Figure 6i–n. The cartilage matrix in the NPR scaffolds was stained by both Alcian blue and toluidine blue. The results verified PGN and GAG generation in the ECM and indicated that the NPR scaffolds could promote mBMSC differentiation into chondrocytes.

**Figure 7:**
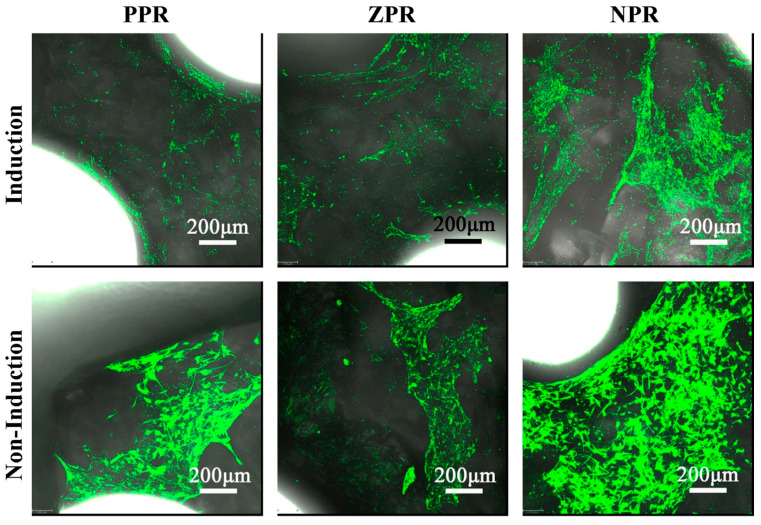
CLSM of mBMSCs induced on CNF/PEGDA scaffolds for 3 weeks.

After induction for three weeks, the scaffolds were stained with HE to observe the morphology and distribution of chondrocytes. In Figure 6, differences in cell morphology can be noted between the non-induced group and the induced group. As shown in Figure 6, cells in the non-induced group tended to grow along the edge of the scaffold, but most of the chondrocytes in the induced group gathered together to form a cell mass. In general, cells tend to aggregate and differentiate in matrices with high porosity [15]. Chondrocytes grow and gather together in scaffolds to form capsular cartilage cells, which can facilitate cell proliferation and differentiation. Comparing the induction effects of the scaffolds with three different Poisson’s ratios in Figure 6 under the same induction time, the honeycomb structure of the scaffolds can affect the differentiation of stem cells. The same conclusion can be drawn from the CLSM diagram after 3 weeks of induction (Figure 7).

The cell morphology analysis shown in Figure 7 revealed that the mBMSCs were scattered on the scaffolds. Compared with the non-induced group, the cells in the induced group were mostly clustered together in clumps. In particular, for the positive Poisson scaffolds, the cells distributed in the non-induced group showed sporadic dispersion and were mostly near the edge of the scaffold, while the cells in the induced group were segmented and linked. This is also different from the fact that the cells in the non-induced group were mainly extended to grow in HE staining, while a large number of cells appeared in the induced group. The observation of cells on scaffolds with different Poisson’s ratios revealed that the cell distribution of the three Poisson’s ratio scaffolds was different. However, how the honeycomb structure of the scaffold affects cell differentiation and whether it affects gene expression still need further study.

Based on the above discussion, aerogel scaffolds could provide a growth environment for mBMSC proliferation and differentiation. Besides, NPR aerogels are more suitable for cell proliferation. There are two main reasons for this phenomenon. First, NPR aerogels better simulate natural tissues and play the role of the ECM to enable normal cell proliferation and aggregation. In addition, NPR aerogels can provide sufficient mechanical support and reserve potential space for tissue development. The macropore aperture of NPR aerogels may be closer to the optimal configuration for cell proliferation and differentiation.

However, the optimum aperture is now in dispute. In tissue engineering, the macropores of scaffolds contribute to the inward growth of stem cells and the transport of nutrients and waste, which can reduce cell accumulation along the edges of the scaffolds. Micropores are conducive to cell attachment and improve the mechanical properties of the scaffold, and the interconnections between small holes improve the porosity of the scaffold to ensure that nutrients can permeate all parts of the scaffold. Studies have found that the aperture size range that promotes chondrocyte proliferation and ECM secretion is 250~500 μm [15]. Figure 3c,f,i show that the macropore aperture in the NPR scaffolds ranged from 400 to 600 μm, which is closer to the optimal macropore aperture than that in the PPR and ZPR scaffolds. This notion is probably why the number of proliferating cells in the NPR scaffolds was the largest during culture. Because the effect of cell proliferation and differentiation depends on the stem cell type, scaffold material and manufacturing conditions, the optimal aperture of CNF/PEGDA aerogel scaffolds in tissue engineering remains to be discussed.

## 4. Conclusions

CNF/PEGDA UV-curing mixtures were used as the ink for SLA. CNF/PEGDA aerogel scaffolds with different Poisson’s ratios were fabricated by combining SLA and freeze-drying. This experimental method provides hierarchical control of the pore structure of nanocellulose scaffolds to obtain clear macropore network structures and interconnected and homogeneously distributed micropores. The present study demonstrated that aerogel scaffolds have a multistage pore structure, including designed macropores and uniformly distributed micropores, with sizes ranging from 20 to 100 μm. In addition, aerogel scaffolds with PPRs, NPRs and ZPRs were realized. Cell culture experiments showed that the honeycomb structure of the scaffold could affect the proliferation of mBMSCs. Alcian blue staining indicated that stem cells on the NPR scaffold were induced to secrete proteoglycans, a chondrocyte characteristic marker. From morphological and quantitative analyses of the cells after induction, we found that NPR scaffolds could provide a growth environment for stem cell proliferation and differentiation, which suggests a promising research direction for cartilage repair in tissue engineering.

## Figures and Tables

**Table 1:** Unit geometry of different Poisson’s ratio structures and relevant dimensional parameters.

Serial Number	Poisson’s Ratio	Each Letter Parameter Relation	Inscribed Radius, R (mm)	Side Length, a (mm)	Wall Thickness, b (mm)	Aperture, d (mm)
P-R1.0	Positive	$\begin{array}{l} d=2R-b\\ R=(a/2)\tan\theta \end{array}$	1.0	1.155	1.2	0.824
Z-R1.0	Zero	$d=b-a\sin^{-1}\gamma$	1.0	1.155	0.86	0.489
N-R1.0	Negative	$d=a-b\sin^{-1}\alpha$	1.0	1.155	0.7	0.371

**Table 2:** Formulas of cellulose nanofiber (CNF)/polyethylene glycol diacrylate (PEGDA) photocurable resin mixtures.

Sample	PEGDA (wt%)	CNF (wt%)	Photoinitiator (wt%)	Deionized Water (wt%)
C-0	20.0	0	0.10	79.0
C-1	20.0	1.0	0.10	78.9
C-2	20.0	2.0	0.10	77.9
I-0.05	20.0	1.0	0.05	78.95
I-0.2	20.0	1.0	0.20	78.8
P-10	10.0	1.0	0.10	88.9
P-30	30.0	1.0	0.10	68.9

## Supplementary Materials

The following are available online at https://www.mdpi.com/2079-4991/11/3/603/s1, Figure S1. The effect of incident exposure on the precision of honeycomb structure of CNFs/PEGDA hy-drogels.
