# Supplementary material for: Nanocellulose/PEGDA Aerogels with Tunable Poisson’s Ratio Fabricated by Stereolithography for Mouse Bone Marrow Mesenchymal Stem Cell Culture
Source: Nanomaterials (Basel). 2021 Feb 28;11(3):603. doi: 10.3390/nano11030603 (PMC7997334; doi:10.3390/nano11030603)
Supplement: Supplementary file 1 [file nanomaterials-11-00603-s001.pdf]

nanomaterials

Supporting Information

Nanocellulose/PEGDA Aerogels with Tunable Poisson's Ratio Fabricated by Stereolithography for Mouse Bone Marrow Mesenchymal Stem Cell Culture

The effect of incident exposure on the precision of honeycomb structure of hydrogels

To obtain the clear pore structure CNFs/PEGDA hydrogel in accordance with the experimental design, the exposure energy was adjusted to ensure that the CNFs/PEGDA hydrogels had the structural outlines. The appearance and structural outlines of the CNFs/PEGDA hydrogels were observed with optical microscope to analyze the clarity and precision. According to the theory of ray propagation, the corresponding program was written. Matlab (MathWorks, Matlab2013a, Natick, MA, USA) was used to simulate the actual photocuring process.

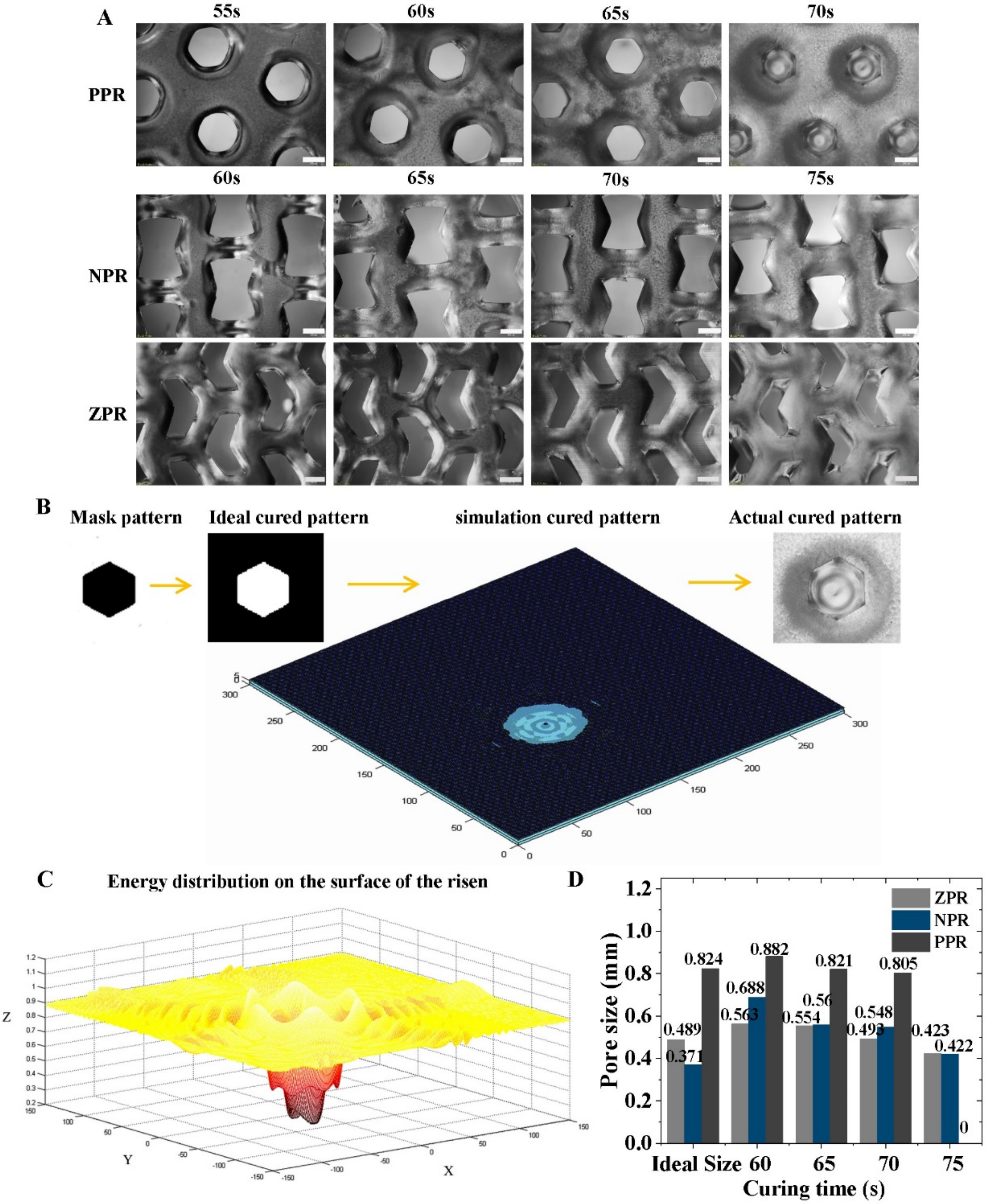

Figure S1. The effect of incident exposure on the precision of honeycomb structure of CNFs/PEGDA hydrogels. (**A**) Optical microscopy of CNFs/PEGDA hydrogel with different grid structures. The radius of the tangential circle (R) is 1.0 mm. (a–d) Negative Poisson's ratio structure;(e–h) Zero Poisson's ratio; (i–l) Poisson's ratio. The scale is 500 μm. (**B**) Comparison of patterns used in

honeycomb structure hydrogel in photocuring experiment: (**a**) mask pattern; (**b**) ideal cured pattern; (**c**) simulation cured pattern and (**d**) actual cured pattern. (**C**) Energy distribution on the surface of the photocuring resin after passing through the mask of honeycomb structure; X and Y axes are the size of the mask pattern (in pixels); Z axis is the normalized light intensity. (**D**) Effect of Curing time on aperture of CNFs/PEGDA hydrogel with different Poisson's ratio.

In Figure S1A, with the increase of curing time, the aperture gradually decreased until it disappears. When the curing time was insufficient, the PEGDA group was not fully crosslinked. However, when the curing time was too long, the PEGDA component was in excessive crosslinking so that the edge of the illuminated area was solidified.

In general, diffraction phenomenon usually occurs when light passes through obstacles. Zhu Qing et al. intuitively simulated the Fresnel parallel lights straight-edge diffraction in Mask Stereolithegraphy through Matlab [1]. Therefore, according to Fresnel diffraction formula, the corresponding program was written in this experiment. The Matlab was used to simulate the curing experiment of mesh structure with positive Poisson's ratio structure in Figure S1B.

The ideal curing pattern should be exactly the same as the pore structure of the mask. Otherwise, diffraction phenomenon occurred when light passes through obstacles or the edge of the opaque part of the mask in the actual photocuring process. There were different degrees of solidification under the opaque part. Comparing simulation cured pattern and actual cured pattern, their shapes were similar. A black dot appeared in the middle of simulation cured pattern, which corresponded to the middle curing point in actual cured pattern. It was due to the diffraction of the light in the middle of the shade, which made the superposition of the light here the strongest, called the Fresnel straight edge diffraction phenomenon. Besides, the over-curing phenomenon was directly related to the distribution of light intensity. After uniform UV light passes through the honeycomb mask, the distribution of light intensity on the resin surface changed due to the diffraction. Figure S1C showed the actual distribution of light intensity. Therefore, it explained the reason why the inner angle of the pore structure was over-cured.

For the PPR hydrogels (Figure S1D), the hydrogel aperture with curing time of 60 s was close to the ideal aperture of 0.824 mm, so the optimal curing time was around 60 s. For the NPR

hydrogels, the hydrogels were cured for 75 s, whose aperture was close to the ideal aperture of 0.371 mm. But there was slight over-curing at the inner Angle of the pore structure. For the ZPR hydrogels, and the aperture closest to the ideal aperture of 0.489 mm was obtained at curing for 70 s, but the inner angle of the pore structure was slightly over-cured. When the curing time is 65 s, the edge of the illuminated area is not solidified. Therefore, the best curing time for ZPR hydrogels was 65~70 s. In summary, the optimal curing duration of positive Poisson's ratio, negative Poisson's ratio and zero Poisson's ratio was around 60 s, 65 s–70 s and 70 s–75 s, respectively.

Reference:

1. Qing Z., Dianhong Y., Analysis of Problem of Fresnel Parallel Light Straight-edge Diffraction in Mask Stereolithegraphy. Tool Eng. **2005**, 6, 74–76.
